# Impregnation of Vitamin D_3_ in *Saccharomyces pastorianus* Cells by Vacuum‐Assisted Biosorption: High Efficiency and Speed Compared to Conventional Method

**DOI:** 10.1111/1750-3841.70576

**Published:** 2025-09-28

**Authors:** Tatielly de Jesus Costa, Marcelo Thomazini, Julia Cristina José, Ramon Peres Brexó, Milena Martelli Tosi, Paulo José do Amaral Sobral, Carmen Sílvia Favaro‐Trindade

**Affiliations:** ^1^ Faculdade de Zootecnia e Engenharia de Alimentos (FZEA) Universidade de São Paulo (USP) Pirassununga São Paulo Brazil

**Keywords:** byproducts, cholecalciferol, micronutrients, vitamin stability, yeast‐based matrices

## Abstract

Vitamin D_3_ (cholecalciferol) deficiency is a major public health challenge worldwide. The development of reliable methods for its protection and distribution is essential for effective supplementation. In this study, vacuum‐assisted biosorption followed by spray drying was investigated to determine the encapsulation of vitamin D_3_ in brewer yeast cells. The size of the particles produced ranged from 20.04 µm (plasmolyzed cells) to 37.40 µm (intact cells), with plasmolyzed cells exhibiting higher electronegativity (zeta potential: −19.8 to −20.0 mV). Morphological analysis using scanning electron microscopy and confocal microscopy revealed greater shrinkage of the plasmolyzed cells. Stability tests over 60 days revealed a 31.2% retention of vitamin D_3_ in intact cells and 20.6% in plasmolyzed cells. The plasmolysis process improved the efficiency of impregnation by about 42%. This method shows potential for stabilizing and protecting vitamin D_3_ in yeast‐based delivery systems, which is a promising approach for sustainable food fortification.

## 1 Introduction

The constant link between food, sustainability, and technological progress has motivated research into new strategies for fortifying foods with important nutrients. Vitamin D_3_ (cholecalciferol) is a micronutrient that has gained importance due to its various health benefits associated with a balanced diet (Fantini et al. 2023). Although several dietary sources of vitamin D_3_ are available, their natural content is generally low and often insufficient to meet the recommended daily intake. This limitation has highlighted the need for efficient strategies to address the nutritional demands of modern society (Vieira and Souza 2022).

The use of industrial waste, such as brewer's yeast biomass, as bio‐vehicle for food fortification represents an excellent opportunity to combine sustainability and nutrition (Coradello and Tirelli 2021). The abundant availability and low cost of *Saccharomyces pastorianus* residual yeast from the brewing industry make it an attractive and environmentally friendly option for this purpose. Its biosorptive properties enable the efficient loading of various compounds, and crucially, cell viability is not a prerequisite for this process, which further supports its large‐scale application (Mitri et al. 2022).

The biosorption technique, which was originally developed for the removal of undesirable compounds, is attracting increasing interest as it is also used for the retention of target compounds. The use of yeast as a biosorbent for bioactive compounds has shown considerable potential and is the subject of current studies on the biosorption of anthocyanins (Nguyen et al. 2022), carotenoids (Rubio et al. 2022), flavonoids (de Andrade et al. 2022), and phenolic acids (Karaman 2021). In a previous study by our research group, *S. pastorianus* biomasses subjected to different treatments were loaded with vitamins C (Jose et al. 2025) and D_3_ (Costa et al. 2024; Soares et al. 2025) by the passive biosorption technique, in which the dispersion was only stirred. Although the process proved to be efficient for loading, it was time‐consuming (300–900 min), which prompted us to look for simpler and faster alternative technologies but equally effective.

Cell pretreatments are commonly used to increase the permeability of yeast structures and thus improve the efficiency of encapsulation of bioactive substances. One of these is plasmolysis, a simple and effective method that promotes the detachment of the plasma membrane by osmotic agents such as NaCl or ethyl acetate (Dimopoulos et al. 2021). As a consequence, the cell becomes considerably more permeable, enabling solutes and solvents to pass through its plasma membrane. This approach has shown positive effects on the incorporation of diverse compounds like plant oils, black cumin, and cholecalciferol (Kavosi et al. 2018; Karaman 2020; Costa et al. 2024). However, efficacy may vary depending on the compound and processing conditions.

In this context, vacuum‐assisted biosorption is a technique based on the application of pressure differences to facilitate the rapid mass transfer of active functional ingredients into porous structures (Saleena et al. 2023; González‐Moya et al. 2023). This technology, an advanced application of Vacuum Impregnation, works through a two‐step pressure change: first, a vacuum is applied; when atmospheric pressure is restored, this pressure differential drives the impregnating solution into the internal spaces of the cells (Saleena et al. 2023). This method has been successfully used to impregnate native *Saccharomyces cerevisiae* cells with curcumin (Young et al. 2020) and to load phenolic‐rich extracts from jabuticaba peels, guarana seeds, and pure catechin into three probiotic strains as biosorbents (Silva et al. 2023).

The advantages of vacuum‐assisted biosorption include its high loading efficiency and the reduced processing time characteristic of this non‐thermal technique (Silva et al. 2023; Saleena et al. 2023). It also allows for controlled and targeted release while improving the storage stability of encapsulated compounds (Silva et al. 2023; Young et al. 2020). Despite these advantages, some limitations remain. The efficacy of biosorption can vary considerably depending on the chemical composition of the compounds involved (Silva et al. 2023; Soares et al. 2025). Moreover, the effectiveness of the process can be strongly influenced by both the compound properties and the processing conditions.

The novelty of this study lies in two main aspects: the sustainable use of *S. pastorianus*, a brewing yeast byproduct, and the application of vacuum‐assisted biosorption. While this method has been investigated with *S. cerevisiae* and probiotic bacteria for compound incorporation (Silva et al. 2023), its use with *S. pastorianus* is still scarcely reported. This is relevant because *S. pastorianus* is an abundant and low‐cost residue from the brewing industry, offering a way to add value to a by‐product that is often underutilized. Combining vacuum‐assisted biosorption with spray drying also provides a promising approach to enrich yeast biomass with bioactive compounds such as vitamin D_3_. The vacuum process enhances compound incorporation into the biological matrix and facilitates specific interactions between the bioactive compound and the biosorbent (Ribeiro et al. 2021; Young et al. 2017). Overall, this strategy increases efficiency, reduces compound degradation, and ensures both cost‐effectiveness and microbiological stability of the final encapsulated product.

The objective of this study was to evaluate the application of vacuum‐assisted biosorption followed by spray drying for the encapsulation of vitamin D_3_ using *S. pastorianus* biomass as a carrier. Specifically, the study assessed the efficiency of vitamin D_3_ incorporation, characterized the resulting microparticles, and investigated the stability of the encapsulated vitamin over time.

## 2 Materials and Methods

### 2.1 Materials

Moist residual brewer's yeast (*S. pastorianus*) biomass provided by Cervejaria Hausen Bier (Araras, São Paulo, Brazil) was used for encapsulant agent. Cholecalciferol was purchased from Sigma‐Aldrich (St. Louis, MO, USA) and used as bioactive compound. Sodium chloride was purchased from Labsynth (Diadema, Brazil) and used for the plasmolysis process. Analyses were performed with chromatographic grade methanol and acetonitrile (Panreac, Barcelona, Spain), analytical grade absolute ethanol (Êxodo Científica, Sumaré, Brazil), and ultrapure water (Merck Millipore, Direct‐Q3).

### 2.2 Determination of Vitamin D_3_

Vitamin D_3_ was quantified via high‐performance liquid chromatography (HPLC), following an adapted procedure from Paucar et al. (2016). The chromatographic system consisted of a Shimadzu Prominence (Japan) equipped with an EVO C18 analytical column (150 × 4.6 mm) and a diode array detector. The mobile phase, composed of a 9:1 (v/v) ratio of methanol: acetonitrile, was used at a flow rate of 0.60 mL/min. Detection was performed at 265 nm, with a sample injection volume of 30 µL and a total run time of 8 min. Vitamin D_3_ was identified based on its retention time (5.30 min) and quantified by plotting the area under the curve (AUC) of the corresponding peak.

### 2.3 Preparation of the Biomass

To prepare the biomass for use, a pre‐purification was carried out by repeated washing with distilled water at an approximate ratio of 1:10 (w/v) until the suspended residues were completely removed. The supernatant was then separated by decantation. The total number of washes was determined by visual assessment of the turbidity of the wash water, with a total of five cycles of water change every 24 h. During this washing process, the biomass was kept refrigerated (3°C–5°C) to minimize the likelihood of spontaneous fermentation, as the optimum temperature for *S. pastorianus* growth is normally between 6°C and 15°C.

The washed yeast was dehydrated using a modified procedure according to Rubio et al. (2020). The biomass was diluted in distilled water at a ratio of 1:3 (w/v) and then dried using a spray dryer (model MSD 1.0, Labmaq do Brasil Ltda, Ribeirão Preto, Brazil) with a 1.2 mm diameter nozzle. The drying conditions included an air inlet temperature of 140°C, selected based on previous studies from our group (Rubio et al. 2020) that demonstrated effective yeast dehydration without loss of integrity under these conditions. Additional parameters were a compressor pressure of 0.39 MPa, outlet temperature of 100°C, feed rate of 0.8 L/h, drying air flow rate of 2.5 m/s, and compressor flow rate of 40 L/min. The dried yeast was stored at −20°C in Schott bottles.

### 2.4 Biomass Plasmolysis

The dried biomass was divided into two distinct parts: intact yeast and plasmolyzed yeast. Plasmolysis was performed according to the method proposed by Karaman (2021), by suspending the dry biomass in Erlenmeyer flasks containing a 10% (w/w) sodium chloride (NaCl) solution. The flasks were then placed in an orbital shaker and incubated at 55°C and 180 rpm for a period of 48 h. This pretreatment was applied to improve vitamin D_3_ retention by increasing cell wall permeability. The plasmolyzed yeast was then dried using spray drying, selected for its efficiency and industrial applicability, according to the previously mentioned drying parameters.

### 2.5 Vacuum‐Assisted Biosorption

For vacuum impregnation, 300 mg of intact and plasmolyzed yeast biomass from both treatments were suspended in 9.2 mL of distilled water. Subsequently, 0.8 mL of an ethanolic vitamin solution (1 mg/mL) was added to the suspension. The concentration of the vitamin solution and yeast biomass and the vacuum holding time (40 s) were determined based on preliminary experiments (data not shown) aimed at maximizing vitamin uptake while maintaining the simplicity and reproducibility of the process. The mixture was shaken continuously for 2 min at 1500 rpm using a Heidolph Multi Reax shaker. The yeast cells prepared with cholecalciferol were then sealed in vacuum bags (15 × 20 cm, TecMac). The bags were sealed with a vacuum sealer (TecMac, TM 100) operating at the maximum vacuum level achievable by the system, corresponding to −101.3 kPa (−760 mmHg), with a holding time of 40 s. The materials were then separated by centrifugation (Eppendorf, 5430R) at 6000 rpm for 5 min at 25°C. Room temperature was selected to preserve vitamin stability and align with mild processing conditions. After that, the samples were frozen at −20°C for further spray drying. The control samples underwent the same processes, except for the addition of the vitamin.

### 2.6 Drying of Enriched Yeast

The enriched biomass from both treatments was spray‐dried following a modified method based on Rocha et al. (2012). The material was diluted 1:2 (w/v) in distilled water and dehydrated using a spray dryer (model MSD 1.0, Labmaq do Brasil Ltda, Ribeirão Preto, Brazil) under similar drying parameters as in Section 2.3, except for the inlet temperature, which was set at 120°C. This condition was chosen to minimize potential vitamin D_3_ loss while ensuring efficient drying, since lower inlet temperatures tested in preliminary trials resulted in incomplete dehydration for this spray dryer configuration. The resulting microparticles were kept at −20°C until further characterization.

### 2.7 Determining Impregnation and Retention

The extraction of the vitamin encapsulated in biomass was performed according to the method described by Paucar et al. (2016) with some changes. Samples (70 mg) were treated with 5 mL of methanol in centrifuge tubes and shaken for 2 min. The samples were then placed in an ultrasonic bath at 25°C for 5 min, followed by centrifugation at 6000 rpm for 5 min. The supernatant was collected and used for quantification of vitamin D_3_ by HPLC (as detailed in Section 2.2). Impregnation efficiency (EI) was determined after vacuum application and retention was evaluated after spray drying, as described in Equations (1) and (2), respectively (Czerniak et al. 2015).

(1) $$\begin{array}{ccc} & & \mathrm{EI}\;\left(\%\right)\\ & & =\frac{\mathrm{Vitamin}\;\mathrm{starter}\;\mathrm{mass}\;-\;\mathrm{Superficial}\;\mathrm{vitamin}\;\mathrm{mas}s^{*}}{\mathrm{yeast}\;\mathrm{mass}\;}\\ & & \times \;100 \end{array}$$

(2) $$\begin{array}{ccc} & & \mathrm{Retention}\;\left(\%\right)\\ & & =\frac{\;\mathrm{Vitamin}\;D_{3}\;\mathrm{concentration}\;\mathrm{in}\;\mathrm{the}\;\mathrm{microparticles}}{\mathrm{Vitamin}\;D_{3}\;\mathrm{concentration}\;\mathrm{in}\;\mathrm{the}\;\mathrm{yeast}\;\mathrm{suspension}}\\ & & \times \;100 \end{array}$$

^*^Superficial vitamin mass is defined as the amount of vitamin present on the surface of a material.

### 2.8 Powder Characterization

The characterization of the vitamin D_3_‐enriched powders was performed, including both intact and plasmolyzed yeasts, and control treatments consisting of intact and plasmolyzed yeasts without vitamin D_3_.

#### 2.8.1 Moisture Content and Water Activity of Powders

The moisture content and water activity (Aw) of the powders were determined using specific analytical equipment. For moisture content, an MB25 Halogen analyzer (Ohaus, Switzerland) was used, which operates with infrared radiation and a halogen heating lamp. Water activity was measured at a temperature of 25°C using an Aqualab Series 3TE (Decagon Devices, USA).

#### 2.8.2 Particle Size Analysis

A MAZ3000 laser diffraction instrument (Malvern Instruments, Worcestershire, United Kingdom) was used to determine the particle size distributions of the powders. The data are reported in terms of the mean diameter along the volume distribution (D[4,3]).

#### 2.8.3 Zeta Potential Analysis

A Zetasizer ZS 3600 analyzer (Malvern Instruments, United Kingdom), with a detection angle of 173° and a wavelength of 633 nm, was used to determine the zeta potentials of the samples.

#### 2.8.4 Microparticle Morphology

The characteristics of the particles were analyzed using scanning electron microscopy (SEM) and confocal microscopy. For SEM analysis, samples were fixed to aluminum rods with double‐sided conductive carbon tape and viewed in a Hitachi TM300 tabletop microscope. For confocal microscopy, the samples were prepared according to the method of Rubio et al. (Rubio et al. 2018) and first immersed in Calcofluor White M2R (1 µg/mL) to stain the cell walls. They were then washed with distilled water and immersed in Nile Red solution (1 µg/mL) to stain the lipid bodies. Cell analysis was performed using a Leica TCS SP5 confocal microscope (Leica Microsystems, Germany).

#### 2.8.5 Differential Scanning Calorimetry

The differential scanning calorimetry (DSC) was conducted using a Mettler Toledo XPR2 device (Schwerzenbach, Switzerland) to record the DSC curves of the microparticles. Each sample was weighed and placed in an aluminum pan and subjected to a thermal cycle ranging from −50°C to 150°C at a heating rate of 10°C/min. Nitrogen was used as the purge gas at a flow rate of 30 mL/min and one void pan was used as the reference.

#### 2.8.6 Infrared Spectroscopy

The samples underwent analysis by Fourier transform infrared spectroscopy (FTIR). A Perkin Elmer FTIR Spectrum One spectrometer, fitted with a Universal ATR sampling accessory, was used to obtain the spectra in the mid‐infrared region. Each measurement consisted of 16 scans conducted at a resolution of 4/cm in the range of 4000–550/cm.

### 2.9 Study of Stability

Samples were placed in small, uncovered glass vials. These vials were then kept at 25 ± 2°C inside larger, hermetically sealed glass jars with clamp lids and rubber gaskets. Each jar was designed as a static equilibrium chamber, containing a saturated solution of magnesium chloride (MgCl_2_) to maintain a stable relative humidity of approximately 35%. Samples were taken at specific time intervals (1, 7, 15, 30, 45, and 60 days) to extract the encapsulated material, using the procedure described in Section 2.7. The half‐life of the encapsulated vitamin was determined as described by Rubio et al. (2020).

### 2.10 Data Analysis

Statistical analyses were conducted using the Statistical Analysis System Version 9.4 software. The data were subjected to ANOVA and Tukey's tests at a significance level of 5%.

## 3 Results and Discussion

### 3.1 Efficiency of Vacuum‐Assisted Biosorption and Retention of Encapsulated Vitamin

The efficiency of vitamin encapsulation by vacuum‐assisted biosorption, evaluated prior to the spray‐drying process, ranged from 55.09% to 96.95% across all treatments involving intact and plasmolyzed yeast cells. The corresponding retention values for these samples are also shown in Table 1. Plasmolysis of the biomass resulted in an approximately 42% increase in biosorption/impregnation efficiency compared to untreated yeast. This increase may be attributed to the increase in intracellular volume due to plasmolysis, which promoted the emptying of cell contents and facilitated the diffusion and uptake of the vitamin into the cells during vacuum biosorption. Moreover, plasmolysis may have induced structural rearrangements in the cell wall and membrane, exposing functional groups such as hydroxyl, amino, and phosphate moieties from polysaccharides and proteins (Pinto et al. 2015). These groups may act as potential binding sites for hydrophobic molecules like vitamin D, thereby facilitating stronger interactions and improving the efficiency of the process.

**TABLE 1:** Encapsulation efficiency and retention of vitamin D_3_ in intact and plasmolyzed yeast cells.

Treatment	Impregnation (%)	Retention (%)
Intact biomass	55.09^b^ ± 0.28	68.17^a^ ± 0.44
Plasmolyzed yeast	96.95^a^ ± 0.11	69.55^a^ ± 0.73

*Note*: Equal letters in the same column indicate that there was no significant difference between the treatments (*𝑝* > 0.05).

The findings of Costa et al. (2024) corroborate these results, demonstrating that plasmolysis significantly increases the passive biosorption efficiency of vitamin D_3_ by the yeast *S. pastorianus* from 44.9% to 88.8%. Similarly, Dadkhodazade et al. (2018) reported a comparable increase in biosorption efficiency when loading *S. cerevisiae* cells with cholecalciferol, increasing efficiency from 31.2% to 76.1% after plasmolysis. However, it is important to emphasize that although plasmolysis optimized the incorporation of vitamin D_3_, both studies showed that passive biosorption required a significant amount of time, ranging from 6 to 12 h. In contrast, the vacuum‐assisted biosorption process used in the present study not only achieved high efficiency (96.9%), but also required significantly less time (40 s). The vacuum conditions likely made it easier for vitamin D_3_ to enter the yeast cells by reducing the external pressure, which may have minimized the resistance of mass transfer through the cell wall and thus increased the efficiency of the process.

As the material was in a medium with a high moisture content, drying was required to obtain it in powder form, making it more microbiologically stable and easier to package, transport, market, and use. Spray drying was chosen as the drying method because it is almost instantaneous and continuous. This process usually results in powders with good flowability and low agglomeration.

However, when the vitamin content was determined after spray drying or in spray‐dried particles, it was found that this process resulted in a considerable loss of about 31% in both treatments. These results suggest that part of the biosorbed vitamin may have been concentrated on the yeast surface, making it more susceptible to thermal degradation during the drying process. To reduce this loss, the use of mild processes or lower temperature for drying, such as electrostatic spraying or lyophilization, could provide a more effective solution and better protection against thermal degradation.

### 3.2 Characterization of the Powders Obtained

After confirming vitamin retention through vacuum‐assisted biosorption, the powders were subjected to physicochemical characterization. This step aimed to elucidate how structural and compositional features, such as water activity, particle size, and morphology, could influence the stability and potential functionality of the encapsulated system.

#### 3.2.1 Water Activity (Aw), Moisture, Zeta (ζ) Potential, and Particle Size

Table 2 shows the results for water activity (Aw), moisture content, ζ‐potential, and particle size results for the yeast biomasses. The analysis included both the biomasses encapsulated with vitamins and their corresponding controls.

**TABLE 2:** Water activity (Aw), moisture, ζ‐potential, and particle size of powders obtained via spray‐drying.

Treatment	Aw	Moisture content (%)	ζ‐Potential (mV)	Average particle size (µm)^*^
Intact biomass	0.46^a^ ± 0.06	6.82^b ^± 0.93	−9.9^b^ ± 0.07	30.24^cb^ ± 2.64
Plasmolyzed yeast	0.46^a^ ± 0.08	7.01^b^ ± 0.53	−19.8ª ± 0.81	20.04^d^ ± 1.26
Intact biomass impregnated with vitamin	0.47^a^ ± 0.02	7.92^a^ ± 0.61	−10.1^b ^± 0.35	37.40^a^ ± 1.79
Plasmolyzed yeast impregnated with vitamin	0.48^a^ ± 0.03	8.31^a^ ± 0.79	−20.0^a^ ± 0.49	31.61^bc^ ± 1.39

*Note*: Equal letters in the same column indicate that there was no significant difference between the treatments (*𝑝* > 0.05).

^*^Size expressed as D[4,3].

Aw is an essential parameter for the microbiological stability of powders and has a direct impact on their shelf life and longevity. Values below 0.6 are considered the recommended threshold for ensuring stability (da Rosa et al. 2019). In this study, Aw ranged from 0.46 to 0.48, showing no statistically significant differences between treatments. However, to ensure stability and a longer shelf life of powders, appropriate packaging and storage conditions must be maintained to prevent moisture absorption.

The moisture content ranged from 6.82% to 7.01% for the controls and from 7.92% to 8.31% for the particles containing the vitamin. These values are within the expected range for powders obtained by spray drying and are comparable to those reported by Costa et al. (2024), who reported moisture contents ranging from 6.75% to 8.58% in cholecalciferol‐loaded *S. pastorianus* yeast powders. Similarly, studies by Sultana et al. (2017) and Rubio et al. (2020) showed that the moisture content of *S. cerevisiae* used for encapsulation of flavors and pigments ranges from 5.1% to 9.2%. Low moisture contents in powders are desirable, as increased moisture content can lead to agglomeration of microparticles and formation of clumps. Agglomeration and clumps negatively affect the structure, flowability, and stability of the encapsulated compound.

The observed ζ‐potential values for intact yeast biomass are consistent with the literature, which reports fluctuations between −5 and −12 mV (Bowen and Ventham 1994; Schwegmann et al. 2010). In contrast, the plasmolyzed cells exhibited significantly more negative ζ‐potential values, ranging from −19.8 to −20.0 mV. This suggests that plasmolysis may have promoted alterations in the surface characteristics of the yeast, possibly by exposing and/or reorganizing charged groups in the cell wall, such as residues of denatured proteins, thereby contributing to the observed increase in electronegativity. These changes in surface properties could be related to structural modifications in proteins and polysaccharides, as further suggested by FTIR analyses discussed in Section 3.2.3.

Wang et al. (2022) noted that the destabilization of yeast cell walls is associated with more negative ζ potentials, corroborating the obtained results. Furthermore, the use of NaCl during plasmolysis may have contributed to the retention of Cl^−^ anions on the cell surface, further enhancing the electronegativity of treated cells.

A ζ‐potential equal to or greater than ± 20 mV is typically interpreted as a sign of stability in dispersion systems (Rogowska et al. 2018). Therefore, it can be inferred that the application of plasmolysis contributed to greater impregnation of the cells during vacuum application because repulsive forces kept them farther apart, making them physically more susceptible to the impregnation process.

The average diameter of the yeast cells ranged from 20.04 µm for plasmolyzed cells to 30.24 µm for intact cells (Table 2). Vacuum‐assisted biosorption resulted in a significant expansion of the cells compared with the control, suggesting that the cells were effectively loaded with vitamin and that plasmolysis facilitated greater loading, as reflected in the increased cell size. These results are consistent with the data on impregnation efficiency (Table 1).

For food applications, particles should have diameters less than 100 µm, facilitating their incorporation into products without negatively impacting texture or consumer acceptance. The particle sizes obtained in this study, ranging from 5 to 150 µm, fall within the typical range of microparticles produced by spray drying, as indicated by Favaro‐Trindade et al. (2010).

#### 3.2.2 Morphology of Microparticles

**FIGURE 1:**
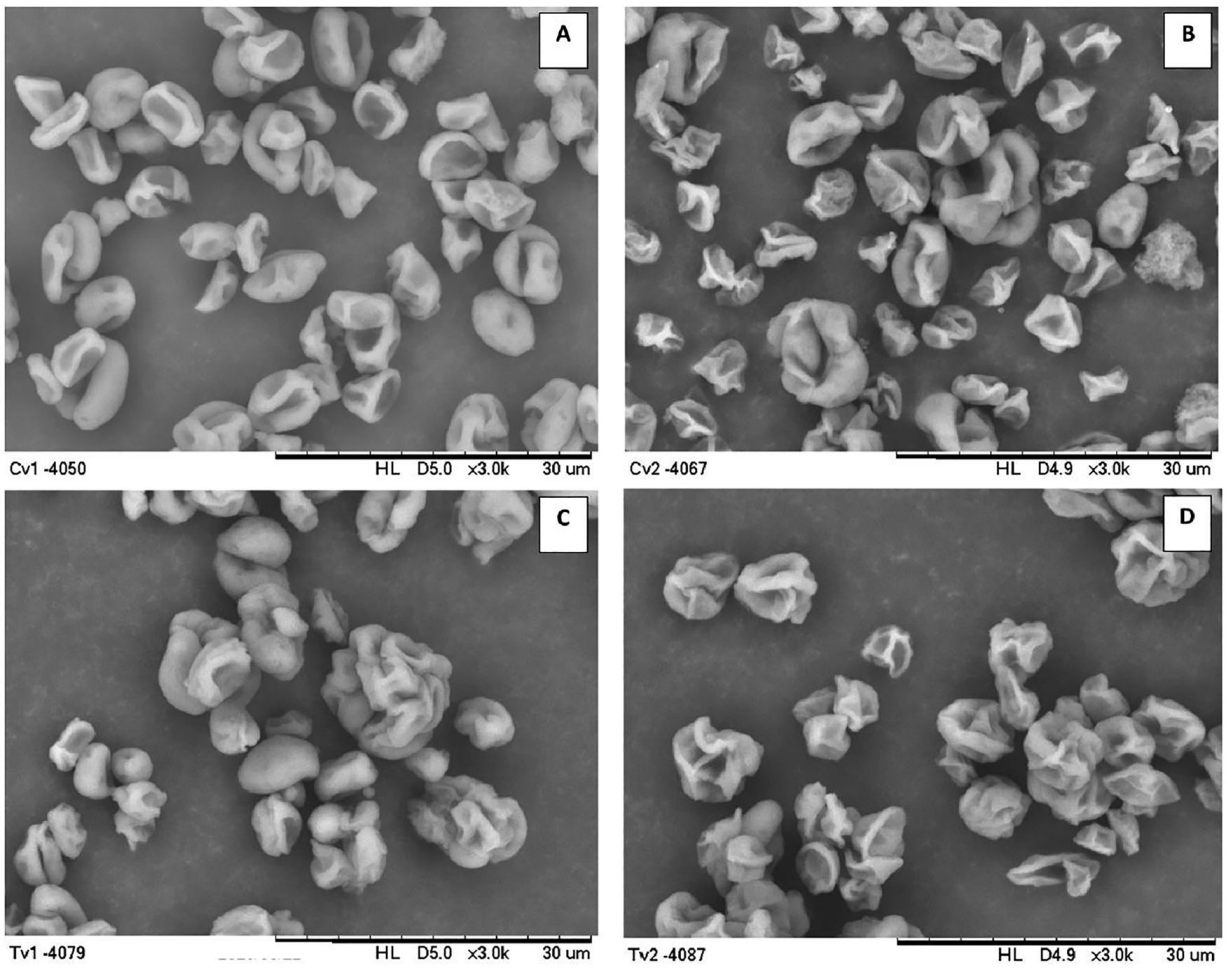
Scanning electron micrographs of intact (A), plasmolyzed (B), intact cholecalciferol‐loaded (C), and plasmolyzed cholecalciferol‐loaded (D) cells.

Scanning electron microscopic analysis of the surface morphology of the yeast cells, both intact and plasmolyzed, revealed concavities and shrinkage in all samples (Figure 1). These effects are likely a result of the spray‐drying process, which, as noted by Favaro‐Trindade et al. (2010), tends to produce microparticles with irregular and contracted surfaces as the water evaporates rapidly from the inside to the outside. In addition, a lower degree of agglomeration was observed in plasmolyzed cells than in untreated cells, which can be explained by the higher electronegativity of these cells (Table 2), which leads to increased electrostatic repulsion.

Morphological changes similar to those observed in the plasmolyzed yeast cells in this study were previously described by Kurek et al. (2023) and Costa et al. (2024). These changes are related to dehydration and cellular contraction caused by hypertonic solvent removal, which can result in the wrinkled and collapsed appearance of the cells. In contrast, the microscopic images of vitamin‐impregnated cells showed slightly greater turgidity than the corresponding controls, suggesting that the vitamin had penetrated the interior of the cells. These observations are supported by data showing an increase in average particle size and higher impregnation efficiency.

**FIGURE 2:**
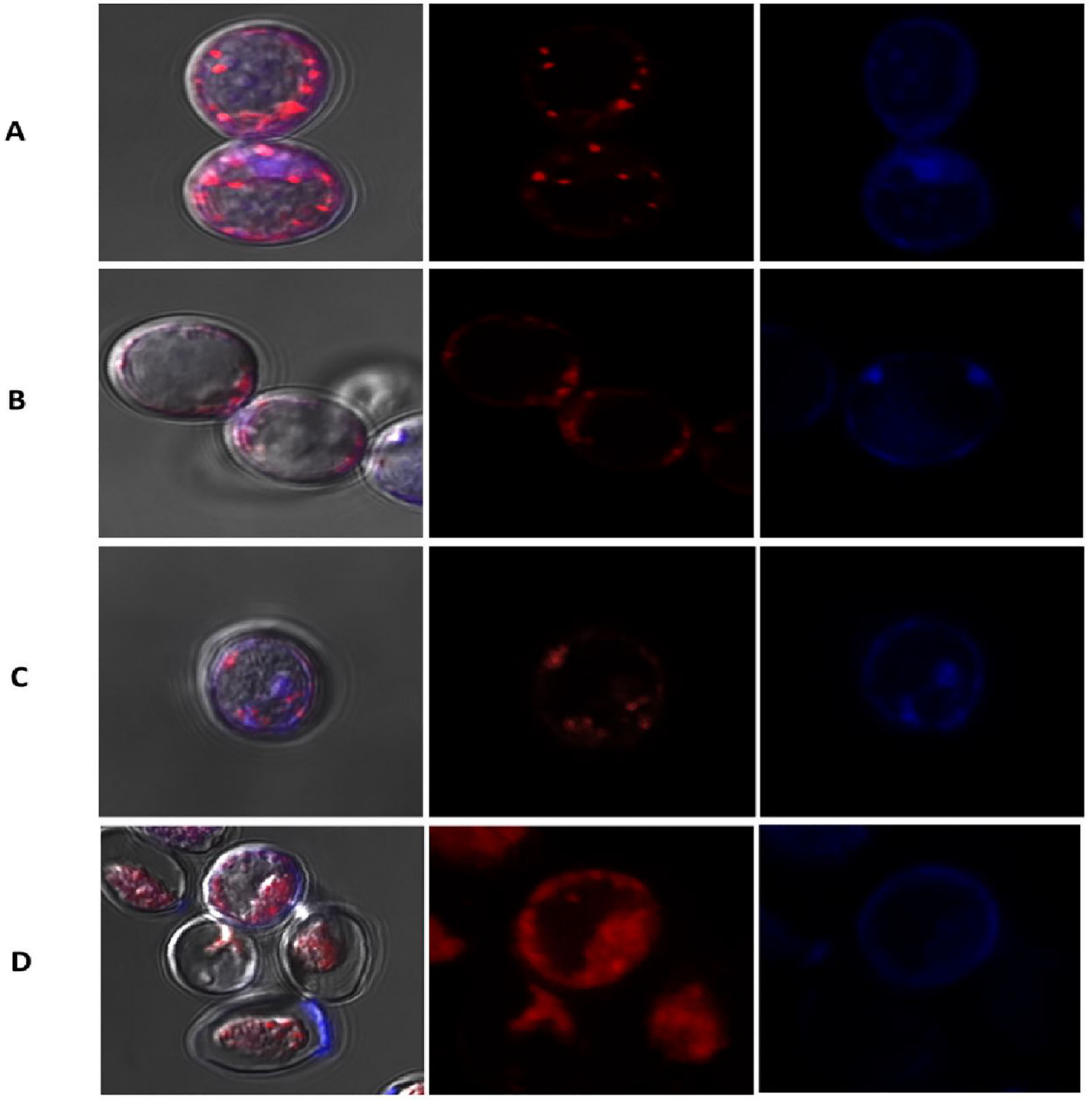
Images of *S. pastorianus* taken with a confocal laser scanning microscope: intact cells (A), plasmolyzed cells (B), intact cells encapsulated with cholecalciferol (C), and plasmolyzed cells encapsulated with cholecalciferol (D). The first column shows microscopic images showing the colors induced by the dyes Calcofluor and Nile Red against a background that does not reveal any specific dye; the middle column shows a contrast that highlights the characteristic red tone of the dye Nile Red; the right column shows the dominance of the blue color typical of the dye Calcofluor.

A morphological analysis was conducted to observe the internal organization and distribution of components within the yeast cells, as depicted in Figure 2. This analysis revealed that cells subjected to plasmolysis showed a higher degree of cell contraction and an accompanying increase in intracellular space. This outcome can be attributed to osmotic dehydration initiated by the plasmolysis process. The observed phenomenon is consistent with findings from previous studies, where plasmolysis was employed as a pretreatment for the encapsulation of oils (Kavosi et al. 2018), gallic acid (Karaman 2021), anthocyanins (Kurek et al. 2023), and cholecalciferol (Costa et al. 2024; Dadkhodazade et al. 2018).

The dye Nile Red was used to identify hydrophobic components, while Calcofluor was used to highlight the main components of the yeast cell wall, such as β‐1,6‐glucans and chitin (Rubio et al. 2020). Excitation of yeast cells with calcofluor resulted in blue fluorescence. Intact control cells showed a more intense blue coloration (Figure 2A), indicating a structurally intact cell wall. In contrast, plasmolyzed control cells showed reduced fluorescence (Figure 2B) and a fragmented appearance of the cell wall, indicating significant structural changes due to plasmolysis.

Yeast cells loaded with vitamin D_3_ exhibited similar staining patterns to the corresponding control groups. With calcofluor, intact cells demonstrated stronger fluorescence (Figure 2C), a finding consistent with the integrity of their cell wall components. Conversely, plasmolyzed cells displayed more intense Nile Red staining, indicating a potential interaction between the vitamin D_3_ and intracellular lipid bodies (Figure 2D). This observation is consistent with the ability of lipophilic compounds to integrate into the lipid bilayers of yeast cells and facilitate their transport into the cytoplasmic space (Kavosi et al. 2018). The red staining was less intense in intact cells but more pronounced in plasmolyzed cells. This difference in red staining intensity, being less pronounced in intact cells but more evident in plasmolyzed cells, suggests that plasmolysis leads to cellular reorganization, possibly promoting the redistribution of lipid components. Similar observations were made by Costa et al. (2024) and Rubio et al. (2020) in studies using confocal microscopy to evaluate cholecalciferol and phenolic compound loading in yeast cells.

#### 3.2.3 Differential Scanning Calorimetry and Fourier Transform Infrared Spectroscopy Studies

**FIGURE 3:**
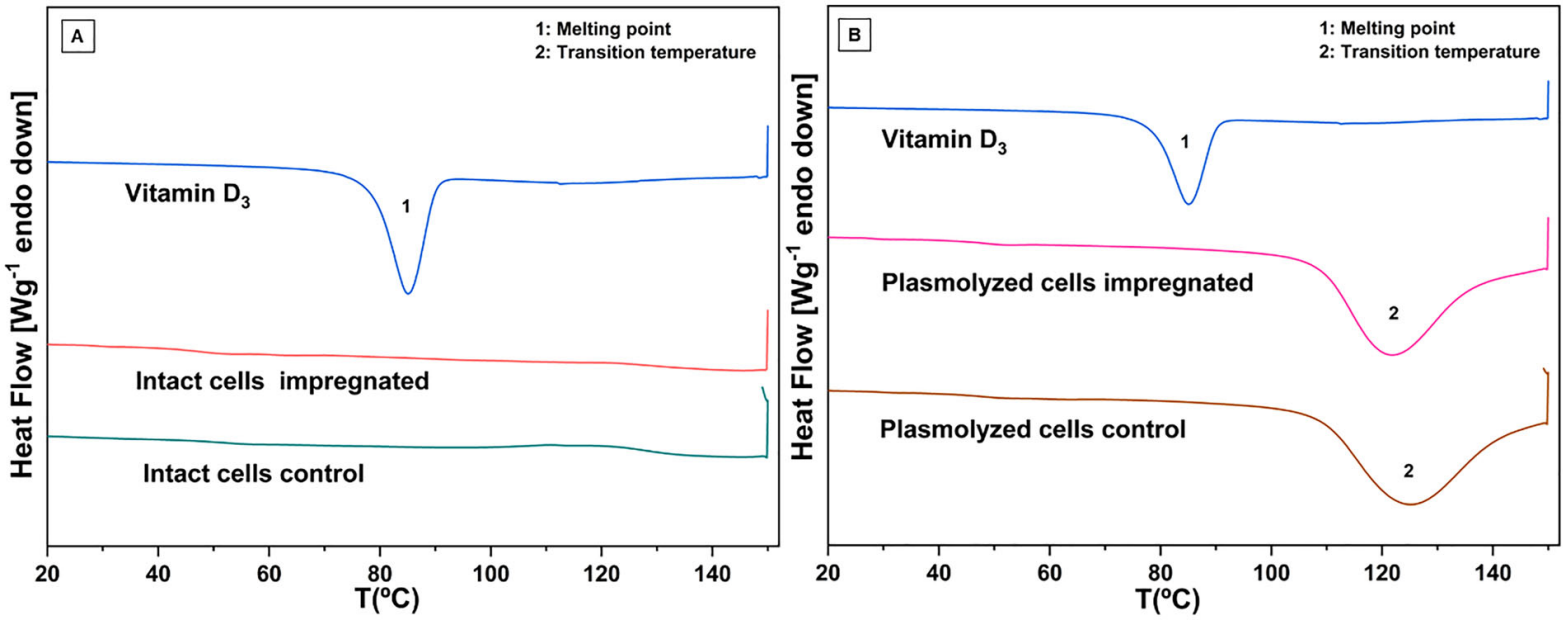
Thermograms: (A) cholecalciferol, intact cells encapsulated with vitamin and intact cells control; (B) Cholecalciferol, plasmolyzed cells encapsulated with vitamin and plasmolyzed cells control.

Yeast cells impregnated with vitamin D_3_, both plasmolyzed and non‐plasmolyzed, were subjected to DSC and FTIR together with the corresponding controls to evaluate possible structural changes due to plasmolysis. DSC analysis enables the detection of endothermic and exothermic transitions as well as changes in the heat capacity of a system over time or temperature. These findings are crucial for the identification of phase transitions, such as glass transition, melting, and crystallization, and provide an in‐depth understanding of the thermal properties and thermal stability of the materials under investigation (Paramera et al. 2011a). The DSC thermograms of vitamin D_3_ and the corresponding yeast biomass samples are shown in Figure 3.

The DSC analysis of cholecalciferol revealed a single endothermic peak at about 85°C due to its melting. And, no phase transitions, even the glass transition, were observed in the DSC curve for intact cells control and intact cells impregned with vitamin D_3_ (Figure 3A). This behavior was certainly due to the amorphous characteristic of these materials which were produced by spray drying. In this process, the drying is quick, not allowing the crystallization of some components, including the vitamin D_3_ which also remained amorphous. Moreover, the absence of an endothermal peak in the yeast‐impregnated samples can suggest that vitamin D_3_ was effectively adsorbed onto the yeast cells, potentially providing protection, and allowing controlled release under specific conditions, which will be investigated in the group's future work.

On another side, a broad endothermic peak was observed in the plasmolyzed yeast samples, with temperatures ranging from 121.3°C in the loaded yeast to 124.2°C in the control yeast. These peaks can be associated to the gel–liquid crystalline transition of the phospholipid bilayer from plasmolyzed yeast, as observed by Paramera et al. (2011a). And this phenomenon occurred in a lower temperature for impregnated samples, probably due to a plasticizer effect of the vitamin D_3_ or as observed by Bondar and Rowe (1995), the vitamin D_3_ introduced a perturbation on phospholipids provoking this reduction in the transition temperature.

Finally, the occurrence of the endothermal phenomenon in the plasmolyzed samples and not in the intact cells can be attributed to the effect of the plasmolysis on the phospholipid composition and/or organization of the yeast plasma membrane, destabilizing it and consequently allowing the phase transition (Ashkezary et al. 2024; Paramera et al. 2011b). This phenomenon was also documented by Normand et al. (2005) and (Semouma et al. 2024), who studied plasmolyzed *S. cerevisiae* for encapsulation of limonene and phenolic compounds from myrtle.

**FIGURE 4:**
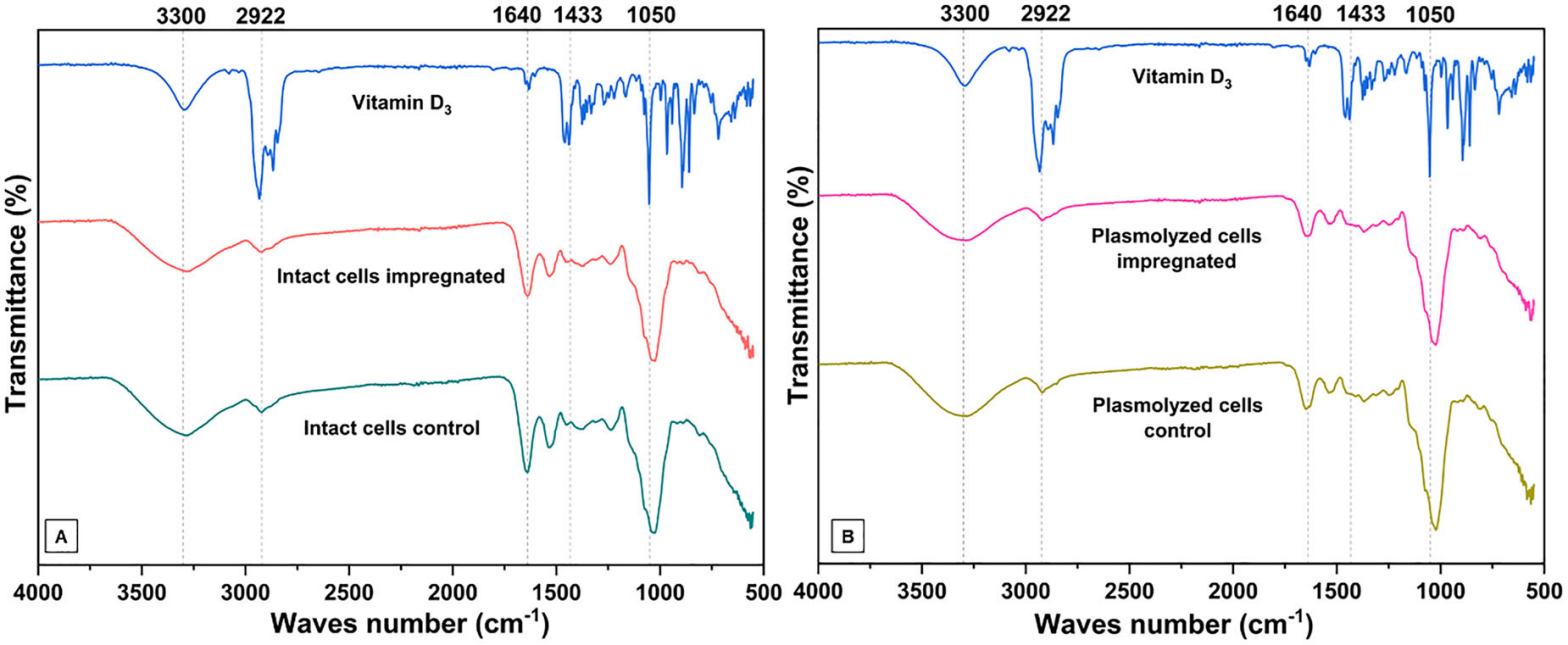
FT‐IR spectra: (A) cholecalciferol, intact cells encapsulated with cholecalciferol and intact cells control; B) Cholecalciferol, plasmolyzed cells encapsulated with cholecalciferol and plasmolyzed cells control.

The FTIR spectra, presented in Figure 4, illustrate the results for free vitamin D_3_, yeast cells enriched with the vitamin, and their corresponding controls. Plasmolysis had a noticeable effect on the absorbance peaks in the range 1640–1433/cm. This region is associated with the amide I and amide II bands, which are characteristic of proteins and peptides (Galichet et al. 2001). The plasmolyzed yeast control exhibited a decrease in the vibrational frequency in this range when compared to the intact cells, suggesting that the plasmolysis process caused structural changes in the proteins. These observations agree with those of Costa et al. (2024), Dadkhodazade et al. (2018), and Salari et al. (2013), who also reported disorganization of the plasma membrane and protein denaturation in the yeast cell wall because of plasmolysis.

In the cholecalciferol spectrum, bands at 3300 and 2922/cm corresponding to stretching vibrations of the O─H and C─H groups were observed, as well as bands at 1640 and 1433/cm associated with C═C vibrations. When analyzing the spectra of the loaded cells, the characteristic cholecalciferol peak at 2922/cm was absent, indicating that this vitamin was indeed adsorbed to the cells, which is confirmed by other results such as average size and DSC. In fact, most of the characteristic peaks of the cells overlapped with those of vitamin D_3_. Moreover, the fact that no difference was observed in loaded and non‐loaded cells of FTIR spectra can denote the physical entrapment of the vitamin D_3_ in the biopolymer network (Gani et al. 2021).

According to Galichet et al. (2001), the infrared beam of the spectrometer (FTIR) penetrates about 3 µm deep into yeast cells and provides information about the components of the cell wall. Considering this property and the fact that cholecalciferol is a non‐polar molecule, it can be concluded that it has a higher affinity to the intact yeast cell wall, especially at the bands around 3300 and 2922 /cm, which are related to the lipid composition. In plasmolyzed yeast, plasmolysis may have facilitated the removal of cellular components, allowing the vitamin to penetrate deeper and possibly bind to intracellular organelles, which could explain the decrease in band intensity in the 3300 and 2922/cm regions. This hypothesis is supported by the higher biosorption efficiency and larger cell size observed in plasmolyzed cells.

### 3.3 Study of the Stability

Subsequently, a stability study was conducted to evaluate the behavior of the powders during storage. Monitoring changes in vitamin retention over time is essential to determine shelf life and to assess the suitability of these powders for practical applications.

**FIGURE 5:**
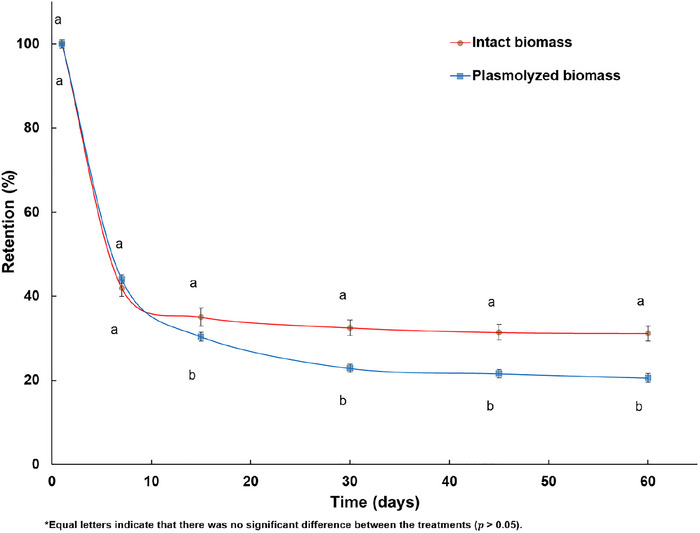
Stability of vitamin D_3_ encapsulated in intact and plasmolyzed yeast cell biomass at 60 days storage, expressed as % retention.

The stability of the encapsulated vitamin was evaluated over a period of 60 days and the results are shown in Figure 5. In the plasmolyzed yeast biomass, the vitamin D_3_ concentration ranged between 2.0 and 0.4 mg/g (dry weight basis), while in the intact cells it ranged between 1.2 and 0.3 mg/g (dry weight basis). The decrease in vitamin concentration, especially during the first 7 days of storage, can be attributed to vitamin degradation. Although the samples were stored in closed vials, residual oxygen trapped during storage or introduced during the high‐temperature atomization step may have initiated oxidative reactions. Furthermore, vitamin molecules adsorbed onto the outermost layers of the yeast cells were more susceptible to degradation, especially under exposure to light and oxygen. Additionally, the porosity of the cell membrane, especially in the plasmolyzed treatment, may facilitate exchange with the external environment, contributing to greater loss of the plasmolyzed cells.

The stability of vitamin D_3_ supplementation is affected by factors such as temperature, oxygen exposure, and light. Previous studies such as those by Abbasi et al. (2014) and Nareswara et al. (2020) indicated that lipophilic vitamins can undergo significant degradation during short storage periods, with retention values of vitamin D_3_ in various matrices such as whey protein and α‐lactalbumin ranging from 16% to 34%. These comparisons suggest that although yeast biomass is subject to degradation, it still offers relatively high vitamin D_3_ retention over a longer period.

In a study by Paucar et al. (2016), the encapsulation of vitamin D_3_ by spray chilling in various lipid matrices led to a retention of 68%–86% after 65 days of storage. Costa et al. (2024) also investigated the stability of cholecalciferol biosorbed by plasmolyzed and non‐plasmolyzed yeast cells and achieved retention rates of 43.73% and 45.85%, respectively, after 60 days of storage. In the present study, 31.2% and 20.6% of vitamin D_3_ were still present in intact and plasmolyzed yeast cells after 60 days, respectively (Figure 5), with half‐lives calculated to be 35.69 and 26.32 days, respectively. Although vitamin D_3_ is degraded over time, the protective effect of yeast cells should not be overlooked. Vacuum‐assisted biosorption in yeast cells, especially those from brewery waste, followed by atomization, continues to provide benefits in terms of vitamin stability and protection during storage. Although a gradual loss of vitamin D_3_ occurred, a significant level of vitamin was maintained with the applied technique, demonstrating the potential of yeast as an effective matrix for stabilizing biosorbed compounds.

## 4 Conclusions

This study demonstrates the effectiveness of vacuum‐assisted biosorption for the impregnation of vitamin D_3_ in *S. pastorianus* yeast cells and emphasizes its high efficiency and speed compared to the conventional method of biosorption. Plasmolysis pretreatment proved to be a crucial strategy to optimize vitamin incorporation, as it induces structural changes in the cell membrane and wall that facilitate the penetration of cholecalciferol. Although the vitamin was retained in the impregnated cells, the results suggest that preservation of the compound during the drying process is a challenge that needs to be overcome.

Thermal and spectroscopic analyses revealed plasmolysis‐induced structural changes in the cells that may have contributed to increased vitamin D_3_ retention. These results suggest that the combination of vacuum‐assisted biosorption is a promising approach to fortify foods with micronutrients such as cholecalciferol. Importantly, employing residual yeast biomass from the brewing industry offers both an effective stabilization matrix and a sustainable strategy by valorizing an abundant industrial byproduct. However, further studies are needed to evaluate the bioavailability and epithelial transport of vitamin D_3_ in the produced microparticles and to assess the impact of this process on various food matrices aiming for practical applications in commercial products.

## Author Contributions

**Tatielly de Jesus Costa**: conceptualization, methodology, formal analysis, writing–original draft, writing–review and editing, investigation. **Marcelo Thomazini**: methodology, validation, data curation. **Julia Cristina José**: investigation. **Ramon Peres Brexó**: methodology, writing–review and editing. **Milena Martelli Tosi**: methodology, data curation. **Paulo José do Amaral Sobral**: data curation, methodology. **Carmen Sílvia Favaro‐trindade**: project administration, funding acquisition, supervision, writing–review and editing, conceptualization.

## Conflicts of Interest

The authors declare no conflicts of interest.

## References

[jfds70576-bib-0001] Abbasi, A. , Z. Emam‐Djomeh , M. A. E. Mousavi , and D. Davoodi . 2014. “Stability of Vitamin D3 Encapsulated in Nanoparticles of Whey Protein Isolate.” Food Chemistry 143, no. January: 379–383. 10.1016/J.FOODCHEM.2013.08.018. 24054255

[jfds70576-bib-0002] Ashkezary, E. Z. , M. Vazifedoost , L. Nateghi , Z. Didar , and M. Moslemi . 2024. “Characterization of Encapsulated Riboflavin in Plasmolyzed and Non‐Plasmolyzed Saccharomyces Cerevisiae Yeast Cells.” Journal of Food Measurement and Characterization 18, no. 6: 4323–4333. 10.1007/S11694-024-02496-9.

[jfds70576-bib-0003] Bondar, O P. , and E. S. Rowe . 1995. “Differential Scanning Calorimetric Study of the Effect of Vitamin D_3_ on the Thermotropic Phase Behavior of Lipids Model Systems.” Biochimica Et Biophysica Acta (BBA)—Biomembranes 1240, no. 2: 125–132. 10.1016/0005-2736(95)00182-4. 8541283

[jfds70576-bib-0004] Bowen, W. R , and T. J. Ventham . 1994. “Aspects of Yeast Flocculation. Size Distribution and Zeta‐Potential.” Journal of the Institute of Brewing 100, no. 3: 167–172. 10.1002/J.2050-0416.1994.TB00817.X.

[jfds70576-bib-0005] Coradello, G. , and N. Tirelli . 2021. “Yeast Cells in Microencapsulation. General Features and Controlling Factors of the Encapsulation Process.” Molecules 26, no. 11: 3123. 10.3390/MOLECULES26113123. 34073703 PMC8197184

[jfds70576-bib-0010] Costa, T. J. , M. Thomazini , J. Cristina José , R. Peres Brexó , M. Martelli‐Tosi , and C. Sílvia Favaro‐Trindade . 2024. “Impact of Plasmolysis Process on the Enrichment of Brewer's Spent Yeast Biomass With Vitamin D_3_ by Biosorption Followed by Spray‐Drying Process.” Food Research International 191: 114677. 10.1016/J.FOODRES.2024.114677. 39059906

[jfds70576-bib-0006] Czerniak, A. , P. Kubiak , W. Białas , and T. Jankowski . 2015. “Improvement of Oxidative Stability of Menhaden Fish Oil by Microencapsulation Within Biocapsules Formed of Yeast Cells.” Journal of Food Engineering 167: 2–11. 10.1016/J.JFOODENG.2015.01.002.

[jfds70576-bib-0007] Dadkhodazade, E. , A. Mohammadi , S. Shojaee‐Aliabadi , A. M. Mortazavian , L. Mirmoghtadaie , and S. M. Hosseini . 2018. “Yeast Cell Microcapsules as a Novel Carrier for Cholecalciferol Encapsulation: Development, Characterization and Release Properties.” Food Biophysics 13, no. 4: 404–411. 10.1007/S11483-018-9546-3.

[jfds70576-bib-0008] da Rosa, J. R. , G. L. Nunes , M. H. Motta , et al. 2019. “Microencapsulation of Anthocyanin Compounds Extracted From Blueberry (Vaccinium Spp.) by Spray Drying: Characterization, Stability and Simulated Gastrointestinal Conditions.” Food Hydrocolloids 89: 742–748. 10.1016/J.FOODHYD.2018.11.042.

[jfds70576-bib-0009] de Andrade, E. W. V. , R. T. Hoskin , and M. R. Da Silva Pedrini . 2022. “Ultrasound‐Assisted Encapsulation of Curcumin and Fisetin Into *Saccharomyces cerevisiae* Cells: A Multistage Batch Process Protocol.” Letters in Applied Microbiology 75, no. 6: 1538–1548. 10.1111/LAM.13820. 36036364

[jfds70576-bib-0011] Dimopoulos, G. , A. Katsimichas , D. Tsimogiannis , V. Oreopoulou , and P. Taoukis . 2021. “Cell Permeabilization Processes for Improved Encapsulation of Oregano Essential Oil in Yeast Cells.” Journal of Food Engineering 294: 110408. 10.1016/J.JFOODENG.2020.110408.

[jfds70576-bib-0012] Fantini, C. , C. Corinaldesi , A. Lenzi , S. Migliaccio , and C. Crescioli . 2023. “Vitamin D as a Shield Against Aging.” International Journal of Molecular Sciences 24, no. 5: 4546. 10.3390/IJMS24054546. 36901976 PMC10002864

[jfds70576-bib-0013] Favaro‐Trindade, C. S. , A. S. Santana , E. S. Monterrey‐Quintero , M. A. Trindade , and F. M. Netto . 2010. “The Use of Spray Drying Technology to Reduce Bitter Taste of Casein Hydrolysate.” Food Hydrocolloids 24, no. 4: 336–340. 10.1016/J.FOODHYD.2009.10.012.

[jfds70576-bib-0014] Galichet, A. , G. D. Sockalingum , A. Belarbi , and M. Manfait . 2001. “FTIR Spectroscopic Analysis of *Saccharomyces cerevisiae* Cell Walls: Study of an Anomalous Strain Exhibiting a Pink‐Colored Cell Phenotype.” FEMS Microbiology Letters 197, no. 2: 179–186. 10.1016/S0378-1097(01)00101-X. 11313132

[jfds70576-bib-0015] Gani, A. , Z. Ul Ashraf , A. Shah , N. Noor , and A. Gani . 2021. “Encapsulation of Vitamin D_3_ Into β‐Glucan Matrix Using the Supercritical Carbon Dioxide.” ACS Food Science and Technology 1, no. 10: 1880–1887. 10.1021/ACSFOODSCITECH.1C00233.

[jfds70576-bib-0016] González‐Moya, S. , A. C. Durán‐Castañeda , R. M. Velázquez‐Estrada , J. A. Sánchez‐Burgos , S. G. Sáyago‐Ayerdi , and V. M. Zamora‐Gasga . 2023. “Vacuum Impregnation of Polyphenols in Yam Bean: Effect on Sensory Acceptability, Antioxidant Capacity, and Potential Absorption Ability.” ACS Food Science and Technology 3, no. 7: 1155–1164. 10.1021/ACSFOODSCITECH.2C00386.

[jfds70576-bib-0017] José, J. C. , B. C. Soares , T. de Jesus Costa , et al. 2025. “Biosorption in Brewer's Spent Yeast Followed by Freeze‐Drying: A Promising Strategy to Protect Vitamin C.” LWT 218: 117494. 10.1016/j.lwt.2025.117494.

[jfds70576-bib-0018] Karaman, K. 2020. “Characterization of *Saccharomyces cerevisiae* Based Microcarriers for Encapsulation of Black Cumin Seed Oil: Stability of Thymoquinone and Bioactive Properties.” Food Chemistry 313: 126129. 10.1016/J.FOODCHEM.2019.126129. 31935665

[jfds70576-bib-0019] Karaman, K. 2021. “Fabrication of Gallic Acid Loaded Yeast (*Saccharomyces cerevisiae*) Microcapsules: Effect of Plasmolysis Treatment and Solvent Type on Bioactivity and Release Kinetics.” LWT 148: 111640. 10.1016/J.LWT.2021.111640.

[jfds70576-bib-0020] Kavosi, M. , A. Mohammadi , S. Shojaee‐Aliabadi , R. Khaksar , and S. M. Hosseini . 2018. “Characterization and Oxidative Stability of Purslane Seed Oil Microencapsulated in Yeast Cells Biocapsules.” Journal of the Science of Food and Agriculture 98, no. 7: 2490–2497. 10.1002/JSFA.8696. 29136285

[jfds70576-bib-0021] Kurek, M. A. , M. Majek , A. Onopiuk , A. Szpicer , A. Napiórkowska , and K. Samborska . 2023. “Encapsulation of Anthocyanins From Chokeberry (*Aronia melanocarpa*) With Plazmolyzed Yeast Cells of Different Species.” Food and Bioproducts Processing 137: 84–92. 10.1016/J.FBP.2022.11.001.

[jfds70576-bib-0022] Mitri, S. , S. J. Salameh , A. Khelfa , et al. 2022. “Valorization of Brewers' Spent Grains: Pretreatments and Fermentation, a Review.” Fermentation 8, no. 2: 50. 10.3390/FERMENTATION8020050.

[jfds70576-bib-0023] Nareswara, A. R. , A. Z. Alamsyah , D. N. Afifah , et al. 2020. “Encapsulation Efficiency of Vitamin D_3_ in α‐Lactalbumin During Storage.” Food Research 4: 141–146. 10.26656/fr.2017.4(S3).S16.

[jfds70576-bib-0024] Nguyen, T. T. , A. Voilley , T. T. T. Tran , and Y. Waché . 2022. “Microencapsulation of *Hibiscus sabdariffa* L. Calyx Anthocyanins With Yeast Hulls.” Plant Foods for Human Nutrition 77, no. 1: 83–89. 10.1007/S11130-022-00947-6. 35072856

[jfds70576-bib-0025] Normand, V. , G. Dardelle , P. E. Bouquerand , L. Nicolas , and D. J. Johnston . 2005. “Flavor Encapsulation in Yeasts: Limonene Used as a Model System for Characterization of the Release Mechanism.” Journal of Agricultural and Food Chemistry 53, no. 19: 7532–7543. 10.1021/JF0507893. 16159183

[jfds70576-bib-0026] Paramera, E. I. , S. J. Konteles , and V. T. Karathanos . 2011a. “Microencapsulation of Curcumin in Cells of *Saccharomyces cerevisiae* .” Food Chemistry 125, no. 3: 892–902. 10.1016/J.FOODCHEM.2010.09.063.

[jfds70576-bib-0027] Paramera, E. I. , S. J. Konteles , and V. T. Karathanos . 2011b. “Stability and Release Properties of Curcumin Encapsulated in *Saccharomyces cerevisiae*, β‐Cyclodextrin and Modified Starch.” Food Chemistry 125, no. 3: 913–922. 10.1016/J.FOODCHEM.2010.09.071.

[jfds70576-bib-0028] Paucar, O. C. , F. L. Tulini , M. Thomazini , J. C. C. Balieiro , E. M. J. A. Pallone , and C. S. Favaro‐Trindade . 2016. “Production by Spray Chilling and Characterization of Solid Lipid Microparticles Loaded With Vitamin D_3_ .” Food and Bioproducts Processing 100: 344–350. 10.1016/J.FBP.2016.08.006.

[jfds70576-bib-0046] Pinto, M. , E. Coelho , A. Nunes , T. Brandão , and M. A. Coimbra . 2015. “Valuation of Brewers Spent Yeast Polysaccharides: A Structural Characterization Approach.” Carbohydrate Polymers 116: 215–222. 10.1016/j.carbpol.2014.03.010. 25458292

[jfds70576-bib-0029] Ribeiro, V. R. , G. M. Maciel , M. M. Fachi , et al. 2021. “Biosorption of Biocompounds From White and Green Tea in *Saccharomyces cerevisiae* Waste: Study of the Secondary Metabolites by UPLC‐QToF‐MS and Simulated In Vitro Gastrointestinal Digestion.” Food Bioscience 41: 101001. 10.1016/j.fbio.2021.101001.

[jfds70576-bib-0030] Rocha, G. A. , C. S. Fávaro‐Trindade , and C. R. F. Grosso . 2012. “Microencapsulation of Lycopene by Spray Drying: Characterization, Stability and Application of Microcapsules.” Food and Bioproducts Processing 90, no. 1: 37–42. 10.1016/J.FBP.2011.01.001.

[jfds70576-bib-0031] Rogowska, A. , P. Pomastowski , M. Złoch , et al. 2018. “The Influence of Different PH on the Electrophoretic Behaviour of *Saccharomyces cerevisiae* Modified by Calcium Ions.” Scientific Reports 8, no. 1: 00. 10.1038/s41598-018-25024-4. PMC5940755 29739986

[jfds70576-bib-0032] Rubio, F. T. V. , C. W. I. Haminiuk , M. Martelli‐Tosi , M. P. Da Silva , G. Y. F. Makimori , and C. S. Favaro‐Trindade . 2020. “Utilization of Grape Pomaces and Brewery Waste *Saccharomyces cerevisiae* for the Production of Bio‐Based Microencapsulated Pigments.” Food Research International 136: 109470. 10.1016/J.FOODRES.2020.109470. 32846555

[jfds70576-bib-0033] Rubio, F. T. V. , C. W. I. Haminiuk , P. D. F. Santos , et al. 2022. “Investigation on Brewer's Spent Yeast as a Bio‐Vehicle for Encapsulation of Natural Colorants From Pumpkin (*Cucurbita moschata*) Peels.” Food & Function 13, no. 19: 10096–11009. 10.1039/D2FO00759B. 36103155

[jfds70576-bib-0034] Rubio, F. T. V. , G. M. Maciel , M. V. da Silva , V. G. Corrêa , R. M. Peralta , and C. W. I. Haminiuk . 2018. “Enrichment of Waste Yeast With Bioactive Compounds From Grape Pomace as an Innovative and Emerging Technology: Kinetics, Isotherms and Bioaccessibility.” Innovative Food Science & Emerging Technologies 45: 18–28. 10.1016/J.IFSET.2017.09.004.

[jfds70576-bib-0035] Salari, R. , B. S. F. Bazzaz , O. Rajabi , and Z. Khashyarmanesh . 2013. “New Aspects of *Saccharomyces cerevisiae* as a Novel Carrier for Berberine.” Daru: Journal of Faculty of Pharmacy 21, no. 1: 73. 10.1186/2008-2231-21-73. PMC3901020 24359687

[jfds70576-bib-0036] Saleena, P. , E. Jayashree , and K. Anees . 2023. “A Comprehensive Review on Vacuum Impregnation: Mechanism, Applications and Prospects.” Food and Bioprocess Technology 17: 1434–1447. 10.1007/S11947-023-03185-Z.

[jfds70576-bib-0037] Schwegmann, H. , A J. Feitz , and F. H. Frimmel . 2010. “Influence of the Zeta Potential on the Sorption and Toxicity of Iron Oxide Nanoparticles on *S. cerevisiae* and *E. coli* .” Journal of Colloid and Interface Science 347, no. 1: 43–48. 10.1016/J.JCIS.2010.02.028. 20381054

[jfds70576-bib-0038] Semouma, D. , I. Laib , D. E. Laib , et al. 2024. “Microencapsulation of *Myrtus communis* Extracts in *Saccharomyces cerevisiae* Cells: Effects on Phenolic Content and Antioxidant Capacity, Physical Characterization and Molecular Docking Analysis.” Food and Bioprocess Technology 17, no. 10: 3281–3304. 10.1007/S11947-023-03316-6.

[jfds70576-bib-0039] Silva, M. P. , R. Rai , C S. Fávaro‐Trindade , and N. Nitin . 2023. “Vacuum‐Assisted Biosorption for Developing Combined Delivery Formulations of Live Probiotics and Plant‐Phenolic Compounds and Their In‐Vitro Evaluation.” Food Bioscience 54: 102732. 10.1016/J.FBIO.2023.102732.

[jfds70576-bib-0040] Soares, B. C. , T. de Jesus Costa , F. L. De Oliveira , et al. 2025. “Vitamin D_3_ Encapsulated in Brewer's Spent Yeast Through Vacuum Biosorption: Studies on the sorption isotherm, release kinetics Into the gastrointestinal system, and colon fermentation.” Food Research International 214: 116597. 10.1016/j.foodres.2025.116597. 40467200

[jfds70576-bib-0041] Sultana, A. , A. Miyamoto , Q. Lan Hy , Y. Tanaka , Y. Fushimi , and H. Yoshii . 2017. “Microencapsulation of Flavors by Spray Drying Using *Saccharomyces cerevisiae* .” Journal of Food Engineering 199: 36–41. 10.1016/J.JFOODENG.2016.12.002.

[jfds70576-bib-0042] Vieira, E. F. , and S. Souza . 2022. “Formulation Strategies for Improving the Stability and Bioavailability of Vitamin D‐Fortified Beverages: A Review.” Foods 11, no. 6: 847. 10.3390/FOODS11060847. 35327269 PMC8955538

[jfds70576-bib-0043] Wang, Y. , P. Zhan , L. Shao , L. Zhang , and Y. Qing . 2022. “Effects of Inhibitors Generated by Dilute Phosphoric Acid Plus Steam‐Exploded Poplar on *Saccharomyces cerevisiae* Growth.” Microorganisms 10, no. 7: 1456. 10.3390/MICROORGANISMS10071456. 35889176 PMC9318740

[jfds70576-bib-0044] Young, S. , S. Dea , and N. Nitin . 2017. “Vacuum Facilitated Infusion of Bioactives Into Yeast Microcarriers: Evaluation of a Novel Encapsulation Approach.” Food Research International 100: 100–112. 10.1016/J.FOODRES.2017.07.067. 28888430

[jfds70576-bib-0045] Young, S. , R. Rai , and N. Nitin . 2020. “Bioaccessibility of Curcumin Encapsulated in Yeast Cells and Yeast Cell Wall Particles.” Food Chemistry 309: 125700. 10.1016/J.FOODCHEM.2019.125700. 31685371

